# The Role of Insula-Associated Brain Network in Touch

**DOI:** 10.1155/2013/734326

**Published:** 2013-07-10

**Authors:** Pengxu Wei, Ruixue Bao

**Affiliations:** ^1^Integrative Rehabilitation Medicine Department, National Rehabilitation Hospital, National Research Center for Rehabilitation Technical Aids, Beijing, China; ^2^Beijing Economic and Technological Development Zone, No. 1 Ronghuazhong Road, Beijing 100176, China; ^3^China Rehabilitation Research Center, Beijing Boai Hospital, School of Rehabilitation Medicine, Capital Medical University, Beijing 100068, China

## Abstract

The insula is believed to be associated with touch-evoked effects. In this work, functional MRI was applied to investigate the network model of insula function when 20 normal subjects received tactile stimulation over segregated areas. Data analysis was performed with SPM8 and Conn toolbox. Activations in the contralateral posterior insula were consistently revealed for all stimulation areas, with the overlap located in area Ig2. The area Ig2 was then used as the seed to estimate the insula-associated network. The right insula, left superior parietal lobule, left superior temporal gyrus, and left inferior parietal cortex showed significant functional connectivity with the seed region for all stimulation conditions. Connectivity maps of most stimulation conditions were mainly distributed in the bilateral insula, inferior parietal cortex, and secondary somatosensory cortex. Post hoc ROI-to-ROI analysis and graph theoretical analysis showed that there were higher correlations between the left insula and the right insula, left inferior parietal cortex and right OP1 for all networks and that the global efficiency was more sensitive than the local efficiency to detect differences between notes in a network. These results suggest that the posterior insula serves as a hub to functionally connect other regions in the detected network and may integrate information from these regions.

## 1. Introduction

Recent studies suggest that touch, as a therapeutic approach, may be effective in treating pain [1] and posttraumatic stress disorder [2], relieving symptoms in patients with cancer [3–5], reducing mortality in patients undergoing percutaneous coronary intervention or elective catheterization [6], and providing psychological support [7]. So and colleagues reviewed randomized controlled trials or controlled clinical trials published before June 2008 to evaluate the effect of touch therapies (Healing Touch, Therapeutic Touch, and Reiki) on any type of pain. They found that touch therapies might have a modest effect on pain relief [1]. Jain and colleagues conducted a randomized controlled trial to determine whether Healing Touch with Guided Imagery could reduce symptoms of posttraumatic stress disorder. Their findings showed that the intervention resulted in a clinically significant reduction in posttraumatic stress disorder and related symptoms [2]. Therapeutic Touch is a safe and beneficial intervention for cancer patients [3]. Aghabati and colleagues examined the effects of Therapeutic Touch, placebo, and usual care on the pain and fatigue of the cancer patients undergoing chemotherapy and found that therapeutic touch was more effective in decreasing pain and fatigue than usual care, whereas the placebo group showed a decreasing trend in pain and fatigue scores compared with the usual care group [4]. In a cohort study with 1290 patients, Cassileth and Vickers found that massage therapy was associated with a substantive improvement in cancer patients' symptoms such as pain, fatigue, stress/anxiety, nausea, and depression [5]. Krucoff and colleagues undertook a multicenter, prospective trial with 748 patients undergoing percutaneous coronary intervention or elective catheterisation to determine the effects of music, imagery, and touch therapy (MIT) on in-hospital major adverse cardiovascular events, 6-month readmission or death, 6-month major adverse cardiovascular events, 6-month death or readmission, and 6-month mortality. The results showed that mortality at 6 months was lower with MIT therapy than without MIT therapy [6]. Jones and Glover explored psychological processes underlying touch through the Alexander Technique. They revealed the touch as a nurturing experience which influenced interpersonal and intrapersonal relational processes [7]. Although these groups applied different types of intervention, the basic component of their methods was tactile stimulation.

Tactile stimulation can activate a number of brain areas, including the insular cortex [8–10]. The insular cortex is believed to be associated with both the physiological and psychological effects evoked by touch [11]. Human insular cortex is a highly interconnected structure in the brain [12]. It is involved in a variety of functions such as somatosensory processing [13, 14], auditory-motor integration [15], auditory perception [16], language processing [17], the emotion processing [18–20], subjective feelings [21], and bodily awareness [11]. 

The insular cortex plays an integrative role and links information from diverse functional systems including social emotional, the sensorimotor, the olfactogustatory, and the cognitive network of the brain [10]. Since a single brain region can exert different functional effects depending on task-dependent network connections, the network connectivity analysis in functional neuroimaging studies has been emphasized [22]. Functional imaging studies in humans have revealed a functional differentiation of the insular cortex and the existence of insula-associated brain network. For example, two recent studies on resting state connectivity [20, 23] found that the anterior insula is functionally connected to the anterior cingulate cortex, whereas the posterior insula is functionally connected to the primary and secondary motor and somatosensory cortices. 

However, few studies have clearly addressed the functional connectivity of human insula when tactile stimulation is applied. In this functional magnetic resonance imaging (fMRI) study, we applied an emotionally neutral tactile stimulation and focused on the effects of this type of touch on insular functional connectivity.

## 2. Subjects and Methods

### 2.1. Subjects

We recruited 20 right-handed healthy male volunteers aged from 20 years to 39 years, with a mean age of 27 years. Two physicians took medical history and then performed physical examination before fMRI experiments to confirm that no subject had previous major medical conditions. A screening form listing conditions that could affect image quality and/or endanger the safety of subjects during magnetic resonance imaging was read and signed by every subject before the experiment. 

Four acupuncture points in the right leg: namely, ST36 (on the relative proximal part of the lateral lower leg; abbreviated as L-P), ST40 (on the relative distal part of the lateral lower leg: L-D), SP9 (on the relative proximal part of the medial lower leg: M-P), and SP6 (on the relative distal part of the medial lower leg: M-D) were chosen as stimulation areas. Tactile stimulation was applied to each area by brushing the subject's skin back and forth using a sponge, at a frequency of approximately 2 Hz; this method was also used in early studies [24–26]. No unpleasant or pleasant feelings were reported after stimulation. Compared with textures-eliciting pleasant feelings, such as those of a soft brush [27] or velvet [28, 29], or those eliciting unpleasant feelings, such as those of coarse sandpapers [28], the sponge texture is soft but relatively rough. Therefore, it corresponds to an affectively neutral modality.

### 2.2. MRI Data Acquisition

We used a whole body 3T Siemens Magnetom Trio system for MRI scanning. The duration of the fMRI experiment was 510 s plus a lead-in period lasting 14 s. This period consists of 16 rest-stimulation cycles (15 s rest, followed by 15 s stimulation), with an additional 30 s rest period at the end. Each of the leg areas was stimulated four times in a randomized order. Gradient echo images with blood oxygen level dependent (BOLD) contrast were collected (TR = 3,000 ms, TE = 40 ms, flip angle = 90°, field of view = 144 mm × 144 mm, and matrix size = 64 × 64). Thirty 5 mm thick contiguous axial slices were acquired for the whole-brain coverage. T1-weighted images (3D MP-RAGE sequence, TR = 1,600 ms, TE = 2.15 ms, flip angle = 9°, Inversion time = 800 ms, FOV = 256 mm × 256 mm, and matrix size = 256 × 256) were also acquired. 

The ethical committee of the hospital approved the protocol. All experiments were conducted in accordance with the Declaration of Helsinki. Written informed consents from all individuals were obtained.

### 2.3. Data Analysis

The images were analyzed using SPM5 (http://www.fil.ion.ucl.ac.uk/spm/). The functional images were motion corrected, spatially normalized in the Montreal Neurological Institute (MNI) space, resampled to 3.0 mm × 3.0 mm × 3.0 mm voxel size, and then spatially smoothed using a 6 mm full-width half-maximum Gaussian kernel. The fMRI activations in white matter, such as those of the internal capsule [30] and corpus callosum [31–33], are thought to connect to different functional networks in the gray matter regions. Hence, we did not use the gray matter mask in the data analysis. In the group analysis, significant changes in the signal intensity of each of the four conditions (i.e., stimulation of four different leg areas, stimulation versus rest) were determined using the mixed-effects model.

#### 2.3.1. Region-of-Interest (ROI) Determination

An important step for connectivity analysis is to define a seed region within the scope of the insula. We defined the seed region using 3 criteria: (1) the seed region was activated in all stimulation conditions; (2) the seed was located within the scope of the insular cortex; and (3) the scope of the seed region should be independent of our data.

Here, a region-of-interest (ROI) analysis was performed to determine the seed region in the insula. Several studies [9, 27, 28, 34–39] show that somatosensory stimuli, including touch, can activate the insular cortex. Thus, we obtained a strong region-based prediction of the group differences and investigated the insular activations in each of the four contrasts using small volume correction [40] at a threshold of *P* < 0.01 with a 10 mm radius centered on the local maxima of the insular activation. The insular gray matter boundary was defined as the anterior, superior, and inferior limiting sulci, the extreme capsule, and the cerebral spinal fluid [9]. 

An activation cluster in the insula may extend to other brain regions such as the parietal operculum. A number of early reports show that tactile stimulation evokes activation in the parietal operculum [25, 41–45]. The parietal operculum and insula (and their subregions) are spatial neighbors [9]; however, these regions have different cytoarchitectonic features [29, 46]. Therefore, their scopes should be clearly defined, and activations in the insula and the parietal operculum should be clearly differentiated. 

For an activation cluster across the insula and other brain regions such as the parietal operculum, we used the SPM Anatomy toolbox [47] to define the scope of insula and assign BOLD signal changes and to determine which part of the evoked activation located in the insular cortex.

#### 2.3.2. Functional Connectivity Analysis

Data were analyzed using a seed-driven approach with the Conn toolbox [48–51] that was designed to work with both resting state scans and block designs. The toolbox performed the first-level General Linear Model for correlation connectivity estimation, and the second-level random-effect analysis.

After images were preprocessed using SPM5, temporal connectivity correlations between the BOLD signal from the seed region and that at every other brain voxel during the entire acquisition period provided seed-to-voxel connectivity estimations. Before averaging individual voxel data, the waveform of each brain voxel was filtered using a bandpass filter (0.008 < *f* < 0.09) to reduce the effect of low-frequency drift and high-frequency noise. Realignment parameters and main session effects were defined as the first level covariates. The signal from ventricular regions and that from the white matter along with their temporal derivatives were also removed through linear regression. 

We generated temporal connectivity maps for each condition by estimating the correlation coefficient between the seed signal and all other brain voxels. In the second-level analysis, the whole-brain connectivity pattern of each stimulation condition was generated. The magnitude and extent of temporal connectivity were thresholded using a false discovery rate (FDR) correction of *P* < 0.05 for the whole brain volume with a minimum cluster extent of 5 contiguous voxels.

#### 2.3.3. Post Hoc Analysis

To further evaluate the features of the detected insular networks, we performed the post hoc ROI-to-ROI analysis and graph theoretical analysis. Common/overlapped areas of the insula-associated networks for the four stimulation conditions were acquired with inclusive masking, at the threshold of *P* < 0.005 with a minimum cluster extent of 5 contiguous voxels. ROIs were then generated from acquired clusters. 

The correlation coefficients between these ROIs were estimated for each condition with the Conn toolbox in the ROI-to-ROI analysis. Here, we focused on comparing the correlations between the left insula and every other ROI/brain region for each stimulation condition. 

Graph theory is a framework for the mathematical representation of the complex network. According to this theory, brain networks can be described as graphs composed of nodes (brain regions or voxels) and edges (structural or functional links) among the nodes [52]. Here, two basic measures for each node (ROI) within the network, global efficiency and local efficiency, were computed as measures of the connectivity using graph theory. Networks between generated ROIs were created by thresholding the correlation matrix at a published threshold, that is, >0.2 [53]. Global and local efficiency indices were thresholded at *p*-FDR < 0.05 in a two-sided analysis based on correlation scores, for each subject and each condition with the Conn toolbox. For each node (ROI), One-way ANOVA analysis was performed to evaluate whether there were any differences in the global or local efficiency between the four stimulation conditions. We also applied ANOVA analysis to evaluate whether there were any differences in global or local efficiency between nodes within each of the four networks. 

## 3. Results

### 3.1. Seed Region

Insular activations were detected only in the left (contralateral) hemisphere. All activations in the insular gray matter were located posterior to the insular central sulcus, that is, in the posterior region of the insula.

Two voxels in the left insular cortex fulfilled the first two criteria previously proposed for the seed region (see Section 2). These voxels were activated in all stimulation conditions and located within the scope of the insular cortex; their MNI coordinates were [(−37)–(−35), (−19)–(−17), 14–16] and [(−34)–(−32), (−19)–(−17), 17–19]. The Anatomy toolbox assigned these voxels to a granular area of the posterior insula called Ig2. Thus, we chose the left Ig2 as the seed region. Based on a cytoarchitectonic study of human insula [29], the scope of area Ig2 was defined by using the Anatomy toolbox and thus was independent of our data. 

### 3.2. Insular Functional Connectivity

**Figure 1:**
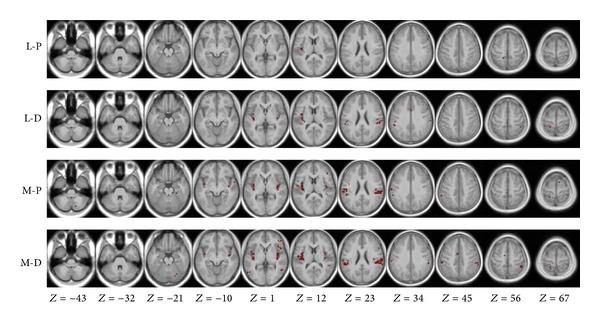
Functional connectivity maps of the stimulated areas. Connectivity maps were projected in an averaged T1 anatomical image of all subjects. The right side of the image corresponds to the right side of the brain.

The connectivity analysis showed that each stimulation condition induced a pattern of functional connectivity (Figure 1). The exact overlap of the four connectivity maps was located in the left insula (one cluster with 73 voxels, acquired via the inclusive masking of SPM software; data was not shown). As another common feature of the four spatial distribution patterns, the significant functional connectivities were revealed between the seed region area Ig2 and the right insula, and left superior parietal lobule, left superior temporal gyrus, as well as left inferior parietal cortex for all stimulation conditions. 

In addition, the functional connectivities were also revealed between the seed and the left OP1, left OP4, right inferior parietal cortex, right inferior parietal lobule, left inferior frontal gyrus, and left middle cingulate cortex for three (L-D, M-P, and M-D) of the four stimulation conditions (see Figure 1 and Table 1). The connectivity maps were mainly distributed in the bilateral insular cortex, secondary somatosensory cortex (subregions OP1–4), and inferior parietal cortex for the three stimulation conditions (i.e., L-D, M-P, and M-D).

**Figure 2:**
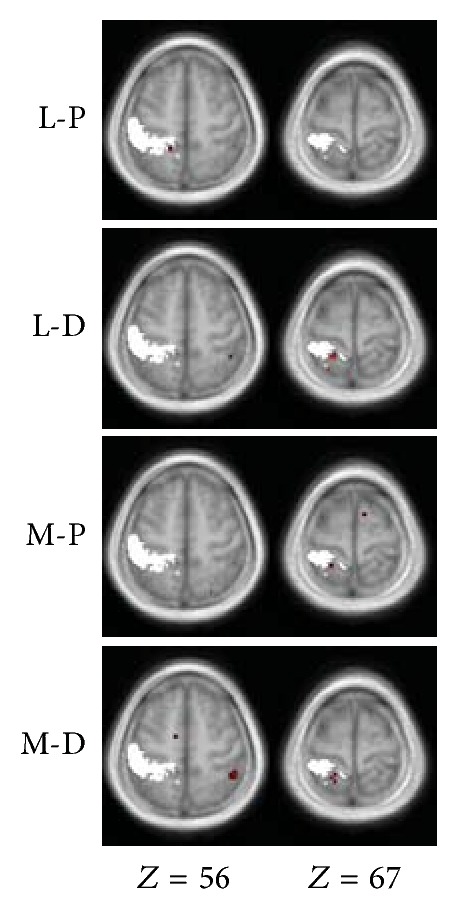
Spatial relations between the primary somatosensory cortex and the correlation map detected in the superior parietal lobule. The right two columns in Figure 1 are shown with the scope of the left primary somatosensory cortex in white. The scope of the left primary somatosensory cortex (summary of subregion areas 1, 2, 3a and 3b) was generated with the SPM Anatomy toolbox.

The locations of clusters detected in the left superior parietal lobule were adjacent to but spatially distinct from the primary somatosensory cortex (Figure 2).

### 3.3. Post Hoc ROI-to-ROI Analysis

**Figure 3:**
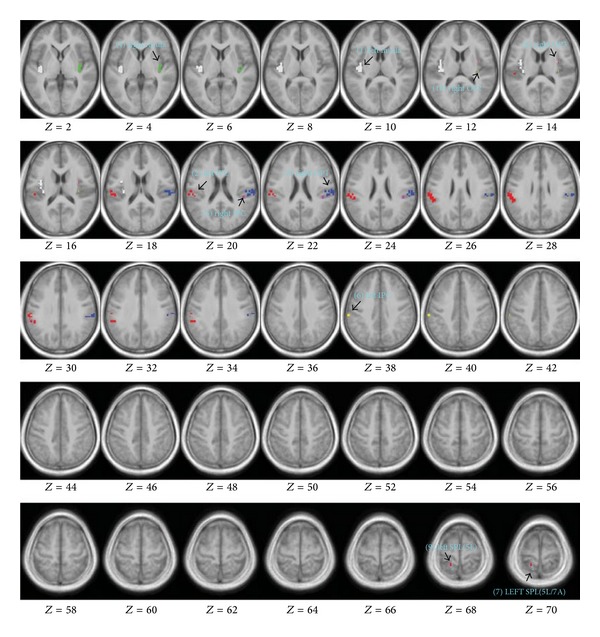
Ten ROIs for the post hoc analysis. Spatial maps of clusters were projected in the averaged T1 anatomical image of all subjects using MRIcron. Numbers in *Z*-axis indicate the MNI coordinate (in mm) defined in SPM software. No. of clusters and the size of clusters are shown in Table 2. Note: (2) left IPC and (6) left IPC had different cluster sizes and spatial locations; and (7) SPL (5L/7A) was also different from (9) SPL (5L).

The common area of the insular networks for the four stimulation conditions was composed of 10 clusters (Table 2). Ten ROIs were then generated accordingly (Figure 3).

Correlations between the left insula and other ROIs were shown in Table 3. The right insula, left inferior parietal cortex, and right OP1 consistently showed stronger correlations with the left insula than other ROIs. The ranks of other ROIs did not show a regular pattern across different stimulation conditions.

### 3.4. Post Hoc Graph Analysis

The global and local efficiency indices at different conditions were listed in Tables 4 and 5, respectively. For each node, no significant statistical differences were observed between the four stimulation conditions. 

For two of the four networks (L-P and L-D), the global efficiency indices between nodes within the network were different (Table 4). By contrast, no significant statistical differences in the local efficiency indices were observed between nodes within each of the four networks (Table 5).

## 4. Discussion

Touches on different body parts or from different genders induce distinct psychological/emotional reactions [54]. In this study, we recruited only male subjects and all examined areas located in a limited lower leg segment. A male physician performed stimulation to all male subjects. Hence, different brain reactions in our results (whether in the insula or other brain areas) were limited to responses to pure somatosensory inputs from the segregated leg areas, without psychological influences from body part or sex differences.

Several studies showed that somatosensory stimuli, whether emotionally neutral, pleasant, or unpleasant, could activate the contralateral midposterior insula [8–10]. The posterior insula was shown to correlate with sensory discriminative functions, whereas the anterior insula is integral in emotional functions [9]. In this study, we applied a neutral touch to nonglabrous skin in four areas of normal subjects. Stimulation over each area evoked only posterior insular activations.

By considering the different functional representations of the face [35], neck [36], shoulder [38], forearm [27, 34, 37, 38], hand [27, 35, 36, 38, 39], leg [34, 37], and foot [35, 39] in the midposterior insula, we conclude that the midposterior insula has a rough topographic representation of all parts of the human body. This representation is consistent with the anatomical topographical projection from thalamic nuclei to the midposterior dorsal insula [8]. By contrast, there is an uncertainty about the localization of somatomotor functions to the insular cortex. A meta-analysis performed by Mutschler and colleagues [15] found that hand and leg motor tasks reproducibly activated the dorsal posterior part of the anterior insula. Nevertheless, it is still unclear whether the activated area is responsible for motor output or for processing sensory information related to the limb movement.

Two recent fMRI studies in humans explored insula-associated brain networks during resting state [20, 23]. Taylor and colleagues [20] focused on the functional connectivity between the insula and cingulate cortex. They found that both the anterior insula and midposterior insula were connected with the posterior midcingulate cortex, whereas the anterior insula was also functionally connected with the pregenual anterior cingulate cortex/anterior midcingulate cortex. Deen and colleagues [23] also found the functional connectivity between the anterior insula and anterior cingulate cortex though they divided the insular in another way (three subregions: the posterior region, dorsal anterior to middle region, and ventral anterior region). The posterior insula was shown to be functionally connected with primary and secondary somatomotor cortices.

### 4.1. Spatial Features of Detected Insular Networks

In our results, the stimulation of segregated body areas evoked distinct functional connectivity maps (Figure 1 and Table 1). For all stimulation conditions, the ipsilateral (right) insula, left superior parietal lobule, left superior temporal gyrus, and left inferior parietal cortex represented a significant functional connectivity with the seed region (left Ig2). This common feature of the four spatial distribution patterns indicates that these brain regions work together to constitute a basic network to process tactile inputs. Furthermore, the connectivity maps of all stimulation conditions (except L-D) were mainly distributed in the bilateral insula, inferior parietal cortex, and subregions of the secondary somatosensory cortex (subregions OP1–OP4), suggesting a major role of these brain regions in the observed insular connectivity network.

During the resting state, the posterior insula is functionally connected to the primary and secondary motor and somatosensory cortices [23]. However, our result showed that no parts of the primary somatosensory cortex are involved in the detected insular connectivity network related to touch. The correlations detected in the superior parietal lobule were spatially outside the primary somatosensory cortex (Figure 2). The primary somatosensory cortex is engaged in the processing and encoding of the type and intensity of the sensory input, whereas the secondary somatosensory cortex processes high-order features of the stimulus such as in the context of attention [55]. Hence, the detected network in this study is obviously not responsible for a discriminative function to process tactile input (as that of the primary somatosensory cortex). This network is also not related to the interoception, a sense of the physiological condition of the entire body, since it is the right anterior insula, a brain region outside this network, that provides the basis of such function [11]. 

All major components in this network exert functions related to touch. Several reports found that tactile stimulation activates the primary auditory cortex located in the superior temporal gyrus [56, 57], one part of the detected insular network. Activations in the auditory cortex evoked by tactile inputs are thought to subserve processing of audiotactile events that arise during dynamic contact between limbs and environment [57]. The superior parietal lobule, also one component of this insular network, is involved in processing tactile information during object exploration and in tactile object discrimination [58–60]. Another component, the inferior parietal cortex, servers as a node to link the tactile perception and manual construction of object shapes [61]; object-oriented action and object recognition activated human inferior parietal cortex, suggesting that some form of within-object spatial analysis was processed in this region [62]. A study also shows that the superior parietal area is involved in spatial processing of tactile inputs, whereas the inferior parietal regions are involved in tactile feature integration and naming [63]. 

People will pay attention to stimulated locations and touch feelings when they are receiving tactile stimulation. The superior temporal gyrus, the superior parietal cortex, and the left inferior parietal regions can be involved in the top-down or stimulus-driven attention, but these functions are largely lateralized to the right hemisphere [64], unlike what we observed here (in the left side). Further studies are needed to determine whether these regions are related to attentional modulations in the detected network. 

By considering functions of these basic components in the detected insular connectivity network, we propose that the posterior insula serves as a hub to functionally connect other brain regions of this network and plays an integral role in touch processing. 

Besides the similarity mentioned above, the connectivity map of each stimulated area represents distinct spatial features (as showed in Figure 1). For example, the map of L-P only consists of 3 clusters, the least of the four maps. The other three connectivity maps also demonstrate different spatial patterns. Such diversity supports the concept of relatively specific effects of stimulation areas in manual therapies of rehabilitation medicine [65] and acupuncture [66, 67]. Various spatial patterns in the detected insular network might be one of the reasons for distinct therapeutic effects evoked by tactile inputs from segregated body parts.

### 4.2. Quantifying Correlations between the Left Insula and Other Regions in the Insular Network

Results from post hoc ROI-to-ROI analysis showed that the right insula, left inferior parietal cortex, and right OP1 consistently had higher correlations with the left insula than other brain areas, indicating that functional connections between the left insula and these regions were generally stronger in the detected networks. 

### 4.3. Global Efficiency and Local Efficiency of Nodes in the Insular Network

The global efficiency measures the extent of information transmission of a given node with all other nodes in a network, whereas the local efficiency measures the extent of information transmission among the neighbors of the node [52]. Our results of the graph theoretical analysis showed that no significant differences in global and local efficiency were found between the four stimulation conditions for each node, indicating that the communication efficiency between each node and its neighbors or all other nodes in the insular networks was not changed much when different body areas were stimulated. 

When comparing nodes within a given network, our results indicated a similar level of connectivity efficiency between each node and its neighbors for every stimulation condition since no differences in the local efficiency indices were observed (Table 5). By contrast, the global efficiency was more sensitive, and two networks, L-P and L-D, showed differences in this efficiency index between nodes in the network (Table 4). 

### 4.4. Limitations of This Study

We only recruited young male subjects in this study, and thus our results cannot be deterministically extended to other populations such as females and old people.

In this study, we measured the “total” functional connectivity between two brain areas (ROIs) by calculating the correlation coefficients between them. This approach cannot determine the unique contribution of a given source ROI on a target ROI (i.e., unable to control the influences of other additional source ROIs). 

## 5. Conclusion

As a highly interconnected region in the human brain, the exact role that the insular cortex plays in processing tactile information is still not fully understood. In this study, we applied three approaches to explore features of the insular network related to tactile stimulation. First, connectivity maps were estimated, and spatial features of these maps were analyzed. Secondly, correlations between the left insula and other regions of the network were quantified with the post hoc ROI-to-ROI analysis. Finally, graph analysis was applied to show the extent of information transmission between each node and its neighbors or all other nodes in the insular network. Thus, similarities and variances between the networks related to segregated body areas were revealed from different perspectives. 

Up to now, human insular network related to touch is rarely reported. Our results indicate that tactile inputs can modulate the function of multiple brain areas via the insular cortex. The insular cortex and insula-associated brain network may be vital to the changes in brain functions evoked by tactile stimulation. 

## Figures and Tables

**Table 1:** Temporal connectivity correlations with the left Ig2.

Brain regions	Side	L-P				L-D				M-P				M-D			
		MNI (mm)			Peak *T* value	MNI (mm)			Peak *T* value	MNI (mm)			Peak *T* value	MNI (mm)			Peak *T* value
		*X*	*Y*	*Z*		*X*	*Y*	*Z*		*X*	*Y*	*Z*		*X*	*Y*	*Z*	
Insula	L	−36	−20	6	12.44	−40	−14	6	10.08	−36	−18	6	10.59	−36	−20	4	10.06
	R					42	−8	6	7.38	36	−6	10	6.28	36	−18	12	7.33
OP1	L					−52	−26	22	6.51	−52	−20	24	6.64	−46	−26	22	8.54
	R									56	−20	22	6.06	60	−26	24	8.36
OP2	R									32	−26	16	4.16	36	−20	18	7.1
OP3	L					−36	−10	12	4.51								
	R									42	−12	12	5.46				
OP4	L					−60	−6	10	6.16	−58	−12	10	6.12	−60	−2	6	5.22
	R									50	−6	10	4.5	54	−6	10	4.96
Superior temporal gyrus	L	−40	−22	4	6.48	−40	−22	2	7.6	−40	−22	4	6.5	−40	−22	4	7.20
	R									42	−36	12	5.26	50	−42	12	5.23
Middle temporal gyrus	L													−54	−66	−2	4.82
	R									50	−14	−12	4.3	56	−48	4	5.93
Temporal pole	L									−58	10	−2	4.91				
	R									56	4	−8	4.72				
Heschl's gyrus	R									42	−20	4	5.42				
Fusiform gyrus	R									26	−32	−20	4.17				
Inferior parietal cortex	L	−60	−30	28	5.53	−52	−26	28	6.17	−64	−26	28	6.84	−58	−24	18	8.5
	R					56	−30	24	6.09	48	−32	28	6	56	−30	24	10.18
Postcentral gyrus	L					−22	−44	70	7.84	−58	−20	40	5.74	−24	−42	52	4.15
Superior parietal lobule	L	−15	−45	58	5.27	−24	−56	70	4.74	−22	−44	70	4.02	−16	−50	76	5.07
	R									24	−68	58	4.76	44	−44	58	5.45
Precuneus	L									−12	−50	72	4.53	−12	−42	72	3.98
Superior frontal gyrus	L													−16	16	52	4.05
Middle frontal gyrus	R													48	46	6	4.66
Inferior frontal gyrus (p. opercularis)	L					−60	6	6	5.45	−54	6	6	5.21	−54	10	6	6.14
	R													56	10	4	5.73
Inferior frontal gyrus (p. orbitalis)	L									−34	34	−6	4.14				
	R													52	40	−4	3.8
Inferior frontal gyrus (p. triangularis)	R									44	34	10	6.21	48	40	0	5.64
Precentral gyrus	L													−52	6	28	4.57
Supplementary motor area	L													−6	−12	64	5.06
	R									8	4	70	4.05	12	10	66	4.8
Paracentral lobule	L													−12	−38	76	3.87
Middle cingulate cortex	L					0	12	34	5.63	−6	−2	40	5.21	4	−2	48	7.1
Middle occipital gyrus	L													−42	−74	18	5.61
Cerebellum	L													−22	−32	−50	4.24
	R									24	−36	−24	4.1	20	−74	−20	5.79
Thalamus	L													−4	−8	4	6.05
	R													6	−18	6	4.52

The MNI coordination of every voxel with the maximal signal change within each cluster was listed (*p*_FDR_ < 0.05, cluster size ≥5 voxels). The anatomical names and locations of cytoarchitectonic areas were output from the Anatomy toolbox. L: left; R: right.

**Table 2:** Ten clusters in the common area of the four correlation maps.

No. of cluster	Location of cluster	Cluster size (voxels)
1	Left insula	317
2	Left IPC	219
3	Right OP1	161
4	Right insula	99
5	Right IPC	18
6	Left IPC	14
7	Left SPL(5L/7A)	8
8	Right OP3	8
9	Left SPL(5L)	6
10	Right OP2	6

The magnitude and extent of temporal connectivity were thresholded using *P* < 0.005 with a minimum cluster extent of 5 contiguous voxels. Each cluster was numbered according to its size (1 being the largest). IPC: Inferior parietal cortex; SPL: superior parietal lobule.

**Table 3:** Correlations between the left insula and other ROIs.

Condition	No. of ROIs	Location of ROIs	Beta	*r*	*T*	*p*-FDR
L-P	4	Right insula	0.80	0.66	8.21	0.000001
	2	Left IPC	0.63	0.56	5.52	0.000057
	3	Right OP1	0.61	0.54	6.29	0.000022
	8	Right OP3	0.49	0.45	4.80	0.000223
	10	Right OP2	0.44	0.41	3.91	0.001400
	9	Left SPL(5L)	0.44	0.41	3.81	0.001515
	5	Right IPC	0.39	0.37	3.65	0.001928
	7	Left SPL(5L/7A)	0.38	0.36	2.36	0.029182
	6	Left IPC	0.37	0.35	5.52	0.000057

L-D	4	Right insula	0.84	0.69	7.76	0.000002
	2	Left IPC	0.76	0.64	5.93	0.000028
	3	Right OP1	0.68	0.59	7.26	0.000003
	9	Left SPL(5L)	0.56	0.51	5.85	0.000028
	7	Left SPL(5L/7A)	0.55	0.50	4.55	0.000331
	6	Left IPC	0.50	0.46	5.51	0.000046
	8	Right OP3	0.43	0.41	4.11	0.000663
	10	Right OP2	0.44	0.41	3.86	0.001054
	5	Right IPC	0.40	0.38	4.32	0.000475

M-P	2	Left IPC	1.04	0.78	8.89	0.000000
	4	Right insula	0.97	0.75	6.64	0.000007
	3	Right OP1	0.84	0.69	6.71	0.000007
	5	Right IPC	0.68	0.59	6.22	0.000013
	10	Right OP2	0.56	0.51	4.14	0.000625
	6	Left IPC	0.55	0.50	5.01	0.000140
	8	Right OP3	0.53	0.49	4.5	0.000366
	9	Left SPL(5L)	0.48	0.45	4.42	0.000381
	7	Left SPL(5L/7A)	0.49	0.45	3.41	0.001867

M-D	4	Right insula	1.02	0.77	7.67	0.000041
	2	Left IPC	0.87	0.70	10.40	0.000000
	3	Right OP1	0.81	0.67	8.33	0.000000
	5	Right IPC	0.72	0.62	5.49	0.000315
	10	Right OP2	0.55	0.50	5.67	0.000048
	8	Right OP3	0.54	0.49	4.15	0.000613
	9	Left SPL(5L)	0.52	0.48	4.57	0.000315
	6	Left IPC	0.46	0.43	4.16	0.000613
	7	Left SPL(5L/7A)	0.37	0.35	3.76	0.001332

Ten clusters were used as ROIs in the ROI-to-ROI analysis. Each cluster was numbered according to its size (1 being the largest), as in Table 2. The 2nd level beta values represented Fisher-transformed correlation coefficient values. The *r* values were acquired by using inverse Fisher transformation.

**Table 4:** Global efficiency at different conditions in the matrix of the insular network.

Nodes	Conditions										
	(1) left insula	(2) left IPC	(3) right OP1	(4) right insula	(5) right IPC	(6) left IPC	(7) left SPL(5L/7A)	(8) right OP3	(9) left SPL(5L)	(10) right OP2	*P* value
L-P											
Mean	0.90	0.91	0.92	0.90	0.89	0.85	0.90	0.84	0.89	0.86	0. 015
Standard deviation	0.06	0.07	0.06	0.09	0.11	0.07	0.09	0.12	0.11	0.09	
L-D											
Mean	0.94	0.93	0.92	0.89	0.87	0.90	0.92	0.87	0.92	0.86	0. 007
Standard deviation	0.08	0.07	0.08	0.10	0.10	0.08	0.06	0.09	0.06	0.07	
M-P											
Mean	0.93	0.95	0.91	0.91	0.92	0.90	0.89	0.88	0.89	0.89	0.282
Standard deviation	0.06	0.06	0.08	0.07	0.09	0.10	0.10	0.08	0.10	0.08	
M-D											
Mean	0.91	0.94	0.92	0.90	0.92	0.90	0.88	0.89	0.90	0.88	0.532
Standard deviation	0.07	0.08	0.07	0.11	0.08	0.09	0.10	0.10	0.11	0.09	
*P* value	0.325	0.279	0.974	0.897	0.237	0.140	0.538	0.443	0.738	0.365	

**Table 5:** Local efficiency at different conditions in the matrix of the insular network.

Nodes	Conditions										
	(1) left insula	(2) left IPC	(3) right OP1	(4) right insula	(5) right IPC	(6) left IPC	(7) left SPL(5L/7A)	(8) right OP3	(9) left SPL(5L)	(10) right OP2	*P* value
L-P											
Mean	0.91	0.91	0.90	0.90	0.90	0.92	0.90	0.95	0.92	0.91	0. 391
Standard deviation	0.05	0.07	0.08	0.06	0.07	0.06	0.07	0.06	0.06	0.09	
L-D											
Mean	0.88	0.91	0.91	0.87	0.92	0.92	0.91	0.92	0.91	0.93	0. 756
Standard deviation	0.09	0.05	0.05	0.21	0.10	0.06	0.06	0.15	0.08	0.05	
M-P											
Mean	0.92	0.91	0.92	0.93	0.91	0.93	0.92	0.92	0.92	0.92	0.999
Standard deviation	0.05	0.05	0.08	0.06	0.07	0.06	0.06	0.07	0.15	0.06	
M-D											
Mean	0.92	0.91	0.91	0.93	0.89	0.93	0.90	0.91	0.90	0.93	0.901
Standard deviation	0.07	0.07	0.08	0.07	0.15	0.06	0.15	0.09	0.18	0.08	
*P* value	0.268	0.999	0.868	0.404	0.647	0.766	0.837	0.575	0.910	0.749	
